# Mapping neonatal vulnerability using the Small Vulnerable Newborn (SVN) framework—secondary analysis of PRISMA Pakistan study

**DOI:** 10.1016/j.lansea.2025.100535

**Published:** 2025-01-25

**Authors:** Hajra Malik, Nida Yazdani, Sameeta Kumari, Sheikh Asad Jamal, Muhammad Kashif, Azqa Mazhar, Zahra Hoodbhoy

**Affiliations:** Department of Pediatrics and Child Health, Aga Khan University Hospital, Pakistan

**Keywords:** Small Vulnerable Newborns, Neonatal mortality, Pakistan

## Abstract

**Background:**

Despite progress in global neonatal mortality, South Asia continues to lag behind in reducing neonatal deaths. The Small Vulnerable Newborn (SVN) framework has been proposed to integrate preterm birth (PT), small for gestational age (SGA), and low birth weight. However, there is lack of data on the burden and risk factors of SVN in Pakistan, a country which has one of the highest neonatal deaths globally. This study aimed to estimate the incidence of SVN, and identify risk factors among pregnant women in Pakistan.

**Methods:**

This secondary analysis leverages data from PRISMA (Pregnancy Risk Infant Surveillance, and Measurement Alliance)—Pakistan. Women presenting ≤20 weeks gestation and, with birth weights recorded within 72 h post-delivery were analysed. Newborns were classified into categories of SVN. Multinomial and binomial regression models were used to examine associations between maternal characteristics and SVN categories, as well as neonatal mortality.

**Findings:**

The overall incidence of SVN was 46% (n = 771) with Term + SGA being the most common category (n = 461, 27.5%), followed by PT + AGA (n = 210, 12.5%) and PT + SGA (n = 41, 2.5%). Maternal undernutrition (MUAC <23 cm) increased the risk of SVN by 17% (aRR 1.17, 95% CI 1.05–1.31). SVN also emerged as a significant predictor of neonatal mortality, quadrupling the risk (aRR 4.52, 95% CI 2.42–8.46).

**Interpretation:**

This study adds to the growing body of evidence on Pakistan's alarming burden of SVN, with every second newborn at risk. Identification and targeted interventions are imperative to mitigate adverse birth outcomes and optimize child growth and development.

**Funding:**

No funding was received for this secondary data analysis.

**Research in context**

**Evidence before this study**

Neonatal mortality remains a pressing global health challenge, with progress particularly slow in low- and middle-income countries (LMICs) such as Pakistan, Traditionally, neonatal vulnerability has been assessed through individual measures like low birthweight (LBW), preterm birth (PT), and small for gestational age (SGA). The lack of an integrated approach led to the development of the “Small Vulnerable Newborns (SVN)” framework, introduced in a recent series published in *The Lancet*, which consolidates the various factors associated with neonatal mortality in a single term. Global SVN prevalence has been estimated at 26.2%, with South Asia bearing the highest burden at 52.1%, Data on SVNs and associated risk factors from LMICs in South Asia remains scarce, highlighting the need for burden identification in the region to drive targeted interventions.

**Added value of this study**

Our study is one of the first to apply the SVN framework to assess neonatal vulnerability in Pakistan. It provides critical insights into the high incidence of SVNs (46%) in peri-urban communities of Karachi, where Term + SGA was the most common category (27.5%), followed by PT + AGA (12.5%). The proportion of PT + SGA newborns (2.5%), the most vulnerable SVN category, was higher than global estimates. We found that SVNs were associated with a four-fold increase in the risk of neonatal mortality. Additionally, our study identified maternal nutrition and parity as significant predictors of SVN.

**Implications of all the available evidence**

To better address the needs of SVNs and achieve the goals of the Every Newborn Action Plan, it is essential to prioritize comprehensive data collection on newborn metrics, and classify newborns according to SVN categories. Our study highlights the significant burden of SVNs in Pakistan, reflecting the broader regional disparities in newborn care seen across South Asia. Factors such as maternal nutrition play a crucial role in determining newborn vulnerability, emphasizing the need for targeted interventions.

## Introduction

Global efforts in reducing neonatal deaths have seen a plateau over the past decade, with many countries falling short on meeting the targets for Sustainable Development Goals (SDGs) for reducing neonatal mortality rate (NMR) to under 12 deaths per 1000 live births by 2030.^1^ The numbers remain a pressing concern with Sub-Saharan Africa and South Asia accounting for nearly three-quarters of all neonatal deaths.^2^ Despite concerted efforts and investments, the 2022 WHO report highlights 2.3 million neonatal deaths, underscoring the urgent need for intensified efforts.^3^ Challenges such as limited access to quality healthcare services, inadequate resources, and socioeconomic disparities continue to hinder progress in low resource countries such as Pakistan, where NMR of 39 deaths per 1000 livebirths ranks amongst the highest in the world.^4^^,^^5^

For decades, newborn vulnerability has been gauged by low birthweight (LBW), defined as infants born weighing less than 2500 g. LBW typically arises from two main etiologies: preterm birth (PT), occurring before the completion of 37 weeks of gestation, and fetal growth restriction (FGR), where the fetal weight falls below the tenth percentile for their gestational age and sex.^6^ A multi-country study assessing the burden of LBW and PT in six LMICs, found Pakistan to have the highest prevalence, with rates of 21.4% for LBW and 21.8% for PT birth.^7^ Public health initiatives have emphasized on LBW and PT births in isolation. However, this approach overlooks other important parameters like SGA and Large for Gestational Age (LGA), as well as their combined effects on neonatal health. Since these parameters are often interlinked in their etiology and care pathways, a comprehensive strategy targeting all these parameters simultaneously is important. A recent series in the Lancet proposed a unified concept: “*Small Vulnerable Newborn* (SVN)”,^8^ which consolidates various factors such as PT, SGA and LBW, associated with neonatal vulnerability into a single term. The framework can be further classified into six categories: Term + Appropriate for Gestational Age (AGA), Term + SGA, Term + LGA, PT + AGA, PT + SGA, and PT + LGA.^9^ According to Lawn et al., the global prevalence of SVN is 26.2%, with Term + SGA accounting for the highest prevalence (16.3%), and PT + SGA with the lowest prevalence (1.1%).^10^ A pooled country analysis of data from LMICs found that Term + SGA babies had a two-fold increased risk of neonatal death [median relative risk (RR) 2.6, interquartile range (IQR) 2.0–2.9], while PT + SGA had a ten-fold increased risk of neonatal death (median RR 10.4, IQR 8.6–13.9).^9^

The availability of data from only 33% of WHO member states highlights limited evidence to understanding this global burden. While existing literature does appreciate the prevalence of LBW, PT, and SGA individually, the scarcity of data on SVN burden, distribution and care gaps necessitates an in-depth examination, particularly in Pakistan. This study adds to the growing body of literature on SVN, by mapping the burden of neonatal vulnerability in an ongoing pregnancy cohort in Pakistan.^11^ This burden estimation can provide valuable insights to inform evidence-based policy decisions to help strategize plans for short-term and long-term health interventions, and improve overall health of mothers and newborns.

## Methods

### Study design and participants

This secondary analysis leverage data from the Pregnancy Risk Infant Surveillance and Measurement Alliance (PRISMA) cohort, an ongoing prospective cohort study in two peri-urban settlements of Karachi, Ibrahim Hyderi and Rehri Goth.^11^ The original study aims to create a harmonized data set to analyze pregnancy risk factors and outcomes in LMICs, to inform targeted interventions and improve maternal and child health.^11^

### Study procedures and outcomes

As part of the PRISMA study, all consented women received an ultrasound scan at the Primary Health Centers (PHC) established by the Department of Pediatrics and Child Health at The Aga Khan University,^12^ to confirm pregnancy viability and determine gestational age (GA). Trained midwives conducted anthropometric measurements including mid-upper arm circumference (MUAC), collected maternal history, and performed clinical examinations and laboratory investigations throughout pregnancy at predefined intervals. This process included documenting the women's demographic and socioeconomic profiles, past obstetric history and current pregnancy characteristics, such as complications like undernutrition (defined as MUAC <23 cm), anemia (defined as hemoglobin <11 g/dL), gestational hypertension, and hepatitis B and/or C status which may be potential risk factors for SVN.^8^ Relevant neonatal outcomes were also recorded, including the birth weight within 72 h of life, GA at delivery, place and mode of delivery, along with neonatal deaths within 72 h of birth. The study was approved by the Aga Khan University Ethical Review Committee (2022-5920-22763).

For the current analysis, all PRISMA enrolled women between January 2021 and August 2022 with an ultrasound confirmed intrauterine pregnancy at less than 20 weeks of gestation, and those who had a live birth with the neonate's birth weight captured within 72 h of delivery were included in the final analysis. Birth weight percentiles were determined according to INTERGROWTH 21 guidelines, utilizing the web-based INTERGROWTH 21 newborn size calculator.^13^^,^^14^

The primary outcome of this analysis was to assess the distribution and risk factors for the six categories of SVN in the current cohort i.e. Term + AGA, Term + SGA, Term + LGA, PT + AGA, PT + SGA, and PT + LGA.

### Data collection and analysis

Data collection was facilitated through an in-built data collection application which longitudinally capture women's information from the time of pregnancy registration until delivery. All collected information was anonymized and subsequently exported into STATA (version 18) for analysis. Descriptive data analysis ensued, employing frequencies and percentages for categorical data, while median and interquartile range (IQR) were utilized for non-normally distributed quantitative variables. We employed two regression models to examine the association between maternal characteristics and the outcome. SVN categories were merged into a binary variable utilizing binomial regression analysis with SVN as an outcome. To assess the relationship between predictors and SVN categories we performed, multinomial regression model using variables significant at univariate for p-value 0.25. We also assessed the association of the SVN categories with the risk of neonatal mortality, while adjusting for the covariates mentioned above.

### Role of the funding source

No funding was received for this secondary data analysis. The primary pregnancy cohort was funded by the Bill and Melinda Gates Foundation (INV-005716).

## Results

**Fig. 1:**
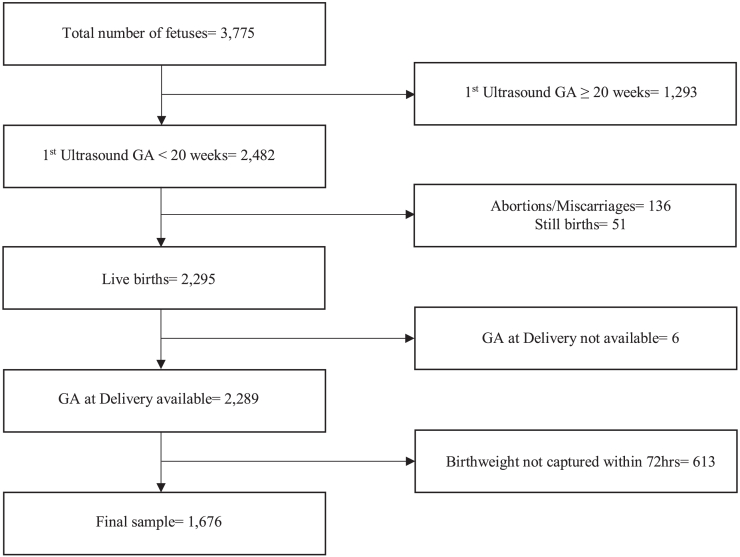
Flow diagram of all the participants included in the final analysis.

Among the 3775 pregnant women enrolled in PRISMA cohort from January 2021 to August 2022, data from 1676 women were included in the final analysis (refer to Fig. 1).

The incidence of SVN in this cohort was 46% (n = 771). In the sub-categories; Term + SGA was most prevalent (n = 461, 27.5%), followed by PT + AGA (n = 210, 12.5%), and PT + SGA (n = 41, 2.5%). The overall incidence of LGA was low, with Term + LGA (n = 36, 2.1%) and PT + LGA (n = 23, 1.4%) accounting for the least common types.

The maternal, labor and delivery characteristics have been described based on the SVN categories in Table 1. Women with PT + SGA neonates had the highest percentage of no formal education (n = 31, 75.6%), tobacco consumption (n = 12, 29.3%), undernutrition (n = 19, 46.3%), and anemia (n = 26, 70.3%). Highest proportion of neonatal deaths were reported in the PT + LGA group (n = 8, 34.8%), followed by PT + SGA (n = 5, 12.2%) group.

**Table 1:** Distribution of maternal and labor and delivery characteristics by six SVN Category (N = 1676).

	Term + AGA	Term + SGA	Term + LGA	PT + AGA	PT + SGA	PT + LGA
	n = 905 (54.0%)	n = 461 (27.5%)	n = 36 (2.1%)	n = 210 (12.5%)	n = 41 (2.5%)	n = 23 (1.4%)
Age at conception mean ± S.D	26.4 ± 5.6	25.8 ± 5.6	29.1 ± 6.9	26.5 ± 5.7	23.8 ± 5.0	27.4 ± 6.0
No formal education	546 (60.3)	279 (60.5)	24 (66.7)	126 (60.0)	31 (75.6)	13 (56.5)
Unemployed	560 (65.1)	291 (66.1)	22 (66.7)	128 (65.6)	21 (52.5)	13 (65.0)
Parity						
Primiparous	176 (19.4)	149 (32.3)	2 (5.6)	40 (9.0)	19 (46.3)	1 (4.3)
Multiparous (2–4)	469 (51.8)	224 (48.6)	22 (61.1)	110 (52.4)	13 (31.7)	17 (74.0)
Grand-Multiparous (≥5)	260 (28.8)	88 (19.1)	12 (33.3)	60 (28.6)	9 (22.0)	5 (21.7)
Bad obstetric history^a^	276 (30.5)	128 (27.8)	8 (22.2)	67 (31.9)	8 (19.5)	8 (34.8)
History of tobacco consumption	199 (22.0)	125 (27.1)	6 (16.7)	56 (26.7)	12 (29.3)	2 (8.7)
Chronic illness^b^	47 (5.2)	28 (6.1)	3 (8.3)	12 (5.7)	1 (2.4)	1 (4.3)
Undernourished (MUAC <23 cm)	208 (23.0)	157 (34.1)	4 (11.1)	54 (25.7)	19 (46.3)	6 (26.1)
Gestational hypertension	37 (4.1)	22 (4.8)	2 (5.6)	12 (5.7)	3 (7.3)	1 (4.3)
Anemia (Hb <11 g/dL)	491 (59.8)	254 (59.9)	21 (63.6)	121 (64.4)	26 (70.3)	10 (45.5)
HBV/HCV reactivity^c^	21 (2.6)	11 (2.6)	1 (3.1)	2 (1.1)	1 (2.6)	1 (4.8)
Mode of delivery						
SVD	637 (70.4)	337 (73.1)	22 (61.1)	118 (56.2)	25 (61.0)	11 (47.8)
C/section	268 (29.6)	124 (26.9)	14 (38.9)	92 (43.8)	16 (39.0)	12 (52.2)
Place of delivery						
Homebirths	100 (11.0)	56 (12.1)	3 (8.3)	28 (13.3)	7 (17.1)	4 (17.4)
Low birth weight	15 (1.7)	249 (54.0)	0 (0)	109 (51.9)	41 (100)	2 (8.7)
Neonatal deaths	12 (1.3)	15 (3.3)	2 (5.6)	17 (8.1)	5 (12.2)	8 (34.8)

a Bad obstetric history = previous history of miscarriage or stillbirths.

b Chronic Illness = Known history of Diabetes Mellitus, Chronic Hypertension, Heart disease, and/or Kidney disease.

c HBV = Hepatitis B Virus, HCV = Hepatitis C Virus.

**Fig. 2:**
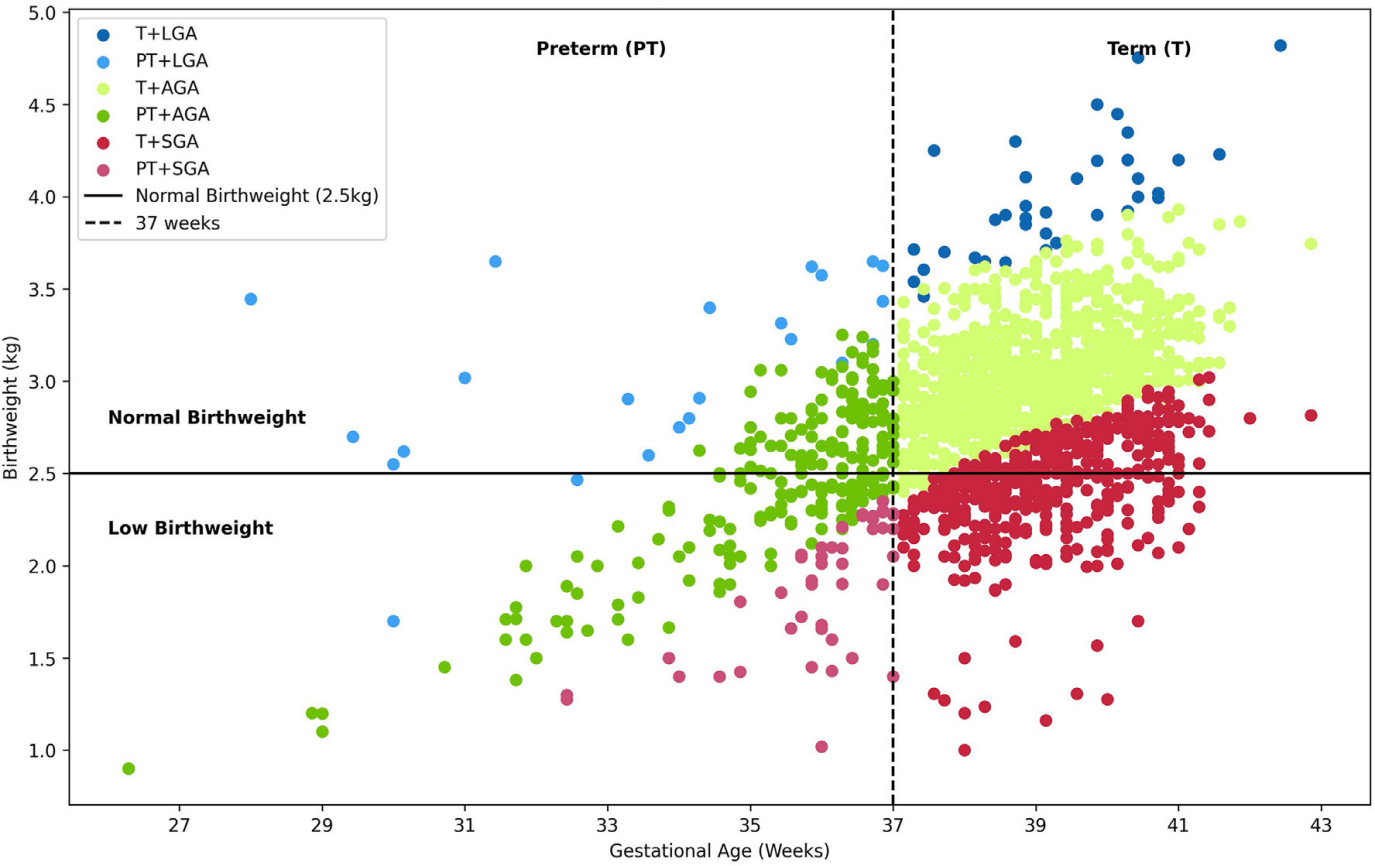
Scatter Plot showing distribution of SVN categories.

We further stratified the six SVN categories according to birth weight status. More than half of the newborns were low birthweight in Term + SGA (n = 249, 54.0%) and PT + AGA (n = 109, 51.9%) groups, while all were low birthweight in the PT + SGA group. Fig. 2 illustrates the distribution of all six SVN categories according to birthweight status.

After accounting for age at conception, number of previous pregnancies, MUAC, gestational hypertension, and tobacco consumption, the number of previous pregnancies decreased risk by 6% per pregnancy (aRR 0.94, 95% CI 0.92–0.97), and undernutrition (MUAC <23 cm) increased risk by 17% (aRR 1.17, 95% CI 1.05–1.31) (refer to Table 2).

**Table 2:** Univariate and Multivariate model for combined SVN births (N = 1676).

	Crude RR (95% CI)	aRR (95% CI)
Age at conception (years)	0.99 (0.98–1.00)^a^	1.00 (0.99–1.02)
No formal education	1.02 (0.92–1.14)	–
Unemployed	1.00 (0.89–1.12)	–
Number of previous pregnancies	0.94 (0.92–0.97)^a^	0.94 (0.92–0.97)^a^
Bad obstetric history^b^	0.95 (0.84–1.06)	–
History of tobacco consumption	1.12 (1.00–1.26)^a^	1.10 (0.98–1.23)
Under-nourished (MUAC <23 cm)	1.24 (1.11–1.38)^a^	1.17 (1.05–1.31)^a^
Anemia (Hb <11 g/dL)	1.04 (0.93–1.16)	–
HBV/HCV reactivity^c^	0.93 (0.64–1.36)	–
Gestational Hypertension	1.14 (0.91–1.42)	–
Chronic illness^d^	1.07 (0.86–1.32)	–

a Maternal and delivery characteristics that showed significant associations (p < 0.25) at univariate and (p < 0.05) at multivariate analysis.

b Bad obstetric history = previous history of miscarriage or stillbirths.

c HBV = Hepatitis B Virus, HCV = Hepatitis C Virus.

d Chronic Illness = Known history of Diabetes Mellitus, Chronic Hypertension, Heart disease, and/or Kidney disease.

Stratified analysis by six SVN categories was performed, controlling for maternal age, number of previous pregnancies, adverse obstetric history, education, employment status, smoking, MUAC, hepatitis B/C status, and anemia which were identified at univariate analysis (refer to Table S1 in the Appendix). Significant variables affecting the different SVN categories were identified through the analysis. For Term-SGA, bad obstetric history increased the risk by 44% (aRR = 1.44, 95% CI: 1.04–2.01), having a MUAC <23 cm increased the risk by 36% (aRR = 1.36, 95% CI: 1.02–1.81), while the number of previous pregnancies reduced the risk by 22% (aRR = 0.78, 95% CI: 0.72–0.85). Each additional year in age at conception increased the risk by 9% for Term-LGA (aRR = 1.09, 95% CI: 1.01–1.16) and reduce it by 13% for PT-SGA (aRR = 0.87, 95% CI: 0.79–0.95). Moreover the risk of PT + SGA increased two-fold for under-nourished (aRR 2.30, 95% CI 1.12–4.77), and uneducated mothers (aRR 2.58, 95% CI 1.03–6.50). Table 3 shows the adjusted risks for each of the SVN categories in detail.

**Table 3:** Multivariate model for six categories of SVN (N = 1339).

	Term + SGA n = 461 (27.5%) aRR (95% CI)	Term + LGA n = 36 (2.1%) aRR (95% CI)	PT + AGA n = 210 (12.5%) aRR (95% CI)	PT + SGA n = 41 (2.5%) aRR (95% CI)	PT + LGA n = 23 (1.4%) aRR (95% CI)
Age at conception (years)	1.02 (0.99–1.05)	1.09 (1.01–1.16)^a^	1.01 (0.98–1.05)	0.87 (0.79–0.95)^a^	1.05 (0.96–1.15)
No formal education	0.93 (0.70–1.22)	1.33 (0.54–3.29)	0.92 (0.63–1.34)	2.58 (1.03–6.50)^a^	1.65 (0.56–4.86)
Unemployed	0.94 (0.72–1.23)	0.86 (0.38–1.95)	0.91 (0.63–1.34)	1.13 (0.55–2.33)	1.27 (0.48–3.38)
Number of previous pregnancies	0.78 (0.72–0.85)^a^	1.02 (0.84–1.24)	0.97 (0.88–1.07)	1.09 (0.87–1.36)	0.82 (0.61–1.12)
Bad obstetric history^b^	1.44 (1.04–2.01)^a^	0.65 (0.25–1.73)	1.23 (0.81–1.87)	0.80 (0.31–2.04)	2.27 (0.74–6.95)
History of tobacco consumption	1.22 (0.89–1.66)	0.73 (0.26–2.01)	1.22 (0.81–1.83)	1.20 (0.55–2.62)	0.35 (0.08–1.59)
Under-nourished (MUAC <23 cm)	1.36 (1.02–1.81)^a^	0.43 (0.12–1.47)	1.03 (0.68–1.54)	2.30 (1.12–4.77)^a^	1.80 (0.65–5.04)
Anemia (Hb <11 g/dL)	1.01 (0.78–1.31)	1.66 (0.73–3.78)	1.33 (0.92–1.90)	1.33 (0.63–2.81)	0.76 (0.29–1.96)
HBV/HCV reactivity^c^	0.98 (0.44–2.17)	0.97 (0.12–8.06)	0.43 (0.10–1.87)	1.04 (0.13–8.20)	2.36 (0.29–19.27)

a Maternal and delivery characteristics that showed significant associations (p < 0.05) at multivariate analysis.

b Bad obstetric history = previous history of miscarriage or stillbirths.

c HBV = Hepatitis B Virus, HCV = Hepatitis C Virus.

We performed an additional analysis to assess the association of the SVN and its categories with the risk of neonatal mortality. After adjusting for age at enrollment, smoking and hypertension status, risk of neonatal mortality increased four times (aRR 4.52, 95% CI 2.42–8.46) amongst all SVN neonates. Further breakdown of the six categories also demonstrated significantly increased risks. PT + LGA newborns faced a 29-fold increased risk (aRR 29.35, 95% CI 14.30–60.24), followed by PT + SGA newborns with an almost 10-fold increased risk (aRR 9.73, 95% CI 3.64–26.01). PT + AGA and Term + SGA newborns also showed significantly higher risks, at 5.7 (aRR 5.73, 95% CI 2.78–11.80) and 2.4 times (aRR 2.41, 95% CI 1.14–5.11), respectively. While, Term + LGA newborns demonstrated increased risk it did not reach statistical significance (aRR 3.81, 95% CI 0.88–16.54). However, these findings must be interpreted in the context of potentially limited precision due to the small sample sizes in each group, as evidenced by the wide confidence intervals.

## Discussion

This study is one of the initial evidence of SVN from a community based study in peri-urban communities of Karachi, Pakistan. The findings revealed an overall high incidence of SVN (46%) with Term + SGA category being the most prominent. Four out of the six vulnerable newborn categories were associated with an increased mortality risk while previous number of pregnancies and maternal nutrition emerged as key predictors of SVN and its categories.

The high burden of SVN in our study (46%) is consistent with global estimates reported for South Asia in general (52.1%),^10^ as well as for individual countries like India (48%).^15^ It provides further evidence of the heightened vulnerability of South Asian newborns as compared to other LMIC settings such as Sub-Saharan Africa (19.9%).^10^ The neonatal profile in South Asia, with Term + SGA reported at 38.8%, diverges sharply from that of Western countries, where PT + nonSGA is the most common SVN category, with a prevalence of 7.2% in North America and Europe.^16^ This regional disparity is mirrored in our study, where Term + SGA accounted for 60% of all SVN. Classification of SVN status is also able to potentially identify more at-risk neonates compared to the conventionally used PT or LBW in isolation. This is evident by the fact that only 50% of Term + SGA neonates in our cohort were LBW. This under-estimation of risk may lead to lack of identification of a vulnerable neonate in a timely manner.

Previous research has emphasized the importance of parity in predicting adverse birth outcomes, with nulliparity being a risk factor.^17^ A study from India found that first-time mothers had a 13% higher risk for SVN (aRR 1.13, 95% CI 1.07–1.20) as compared to multiparous mothers.^15^ Our study mirrors this trend, showing a six percent reduction in SVN risk with each additional pregnancy. This protective effect is more pronounced in the Term + SGA category, where having a previous pregnancy is associated with a 22% lower risk. Literature indicates that primiparous women face heightened risks for adverse birth outcomes, driven by factors ranging from extremes of maternal age, lack of knowledge regarding importance of antenatal care, and/or danger signs during pregnancy.^18^^,^^19^

Maternal nutrition is widely recognized as an important indicator during pregnancy, influencing both maternal and fetal outcomes.^17^ A systematic review from LMICs found that underweight women were 85% more likely to have a SGA birth (OR 1.85, 95% CI 1.69–2.02), 66% more likely to have a LBW newborn (OR 1.66, 95% CI 1.50–1.84), and 13% more likely to have a preterm birth (OR 1.13, 95% CI 1.01–1.27).^20^ Utilizing MUAC as a nutritional indicator, our study showed that undernourished women were at 17% higher risk to have SVN, which further increased two-fold for PT + SGA. Even when using BMI for assessing maternal nutritional status, a similar trend with a 19% increased risk was observed in women with BMI of less than 18.5 kg/m^2^, with PT + SGA again emerging as the highest risk category.^15^ Hofmeyer et al. estimated that implementing the full WHO antenatal care package, including Multiple Micronutrient Supplements (MMS) and balance energy protein (BEP), could prevent up to five million SVN births annually in LMICs.^21^ In addition, the WINGS Trial reported a 15% risk reduction in LBW (IRR 0.85, 95% CI 0.75 to 0.97), while evaluating for the effect of preconception intervention package comprising of health, nutrition, psychosocial care, and WaSH interventions.^22^ These findings highlight the importance of prioritizing maternal nutrition during pre-conception, and the pregnancy period to improve neonatal outcomes.

Maternal anemia is recognized as a major risk factor for SVN.^17^ In our study, although more than half of the women (55%) had anemia at enrollment, it did not emerge as a statistically significant predictor for SVN or its categories. This contrasts with findings from India which reported anemia diagnosed anytime during pregnancy was associated with a 38% increased SVN risk.^15^ This discrepancy could be attributed to anemia solely being assessed at the time of enrollment in the current study.

The proportion of neonatal deaths was highest among the preterms, PT + LGA (34.8%), PT + SGA (12.2%), and PT + AGA (8.1%), as compared to the term infants, emphasizing the gaps in care for preterms and need for improved perinatal interventions. SVN also showed a four times higher risk of neonatal mortality when compared to Term + AGA neonates. Similar to our study's findings, a descriptive analysis from nine LMICs found that besides Term + LGA, all SVN categories significantly increased the risk of neonatal mortality, with ten times higher risk if PT + SGA.^9^ Findings from Bangladesh, further support the vulnerability of SVN newborns with 13-fold increased risk in PT + SGA category for neonatal mortality (HR 13. 25, 95% CI 8.65–20.31).^23^ While most studies report higher neonatal deaths in the PT + SGA group, our study found higher mortality in the PT + LGA group. One possible reason could be the small sample size. Additionally, a multi-country study suggests that using the 90th percentile as a threshold for LGA may not accurately predict neonatal mortality, and higher percentiles (>95 or >97) might be more reliable.^24^ Clinically, LGA neonates are at increased risk of obstructed labor, prolonged labor, and perinatal trauma, which could contribute to the higher mortality.^25^ These numbers press upon the need for a composite and accurate assessment of vulnerabilities in newborns to ensure that such neonates are identified timely for further management. In countries like Pakistan where the burden of neonatal mortality and morbidities is disproportionately high, a comprehensive assessment of vulnerability as provided by the SVN framework can help identify neonates who may require further attention on their growth and development.

The strength of this research stems from its comprehensive analysis of a community based cohort with a high SVN burden, along with accurate gestational age determination through ultrasound assessments within the first 20 weeks of pregnancy. Trained and certified staff performed anthropometric measurements, ensuring reliability and accuracy in assessing weight within 72 h of birth. This is very important in countries like Pakistan where documentation on labor and delivery information is poor. However, this study was conducted in a small setting at peri-urban settlements of Karachi, a relatively homogenous population in terms of socioeconomic status. The data on birth weights of stillborns, information on interpregnancy intervals, and maternal infections during the antenatal period was limited, potentially restricting these to be included in the analysis. Further, there were 27% (n = 613) neonates where birthweight was not available within the first 72 h and hence were excluded from the analysis. This may lead to some reoorting bias.

To better address the needs of SVN and achieve Every Newborn Action Plan, it is essential to prioritize comprehensive data collection on newborn metrics, and classify them according to the vulnerability categories. Our study stresses the high burden of SVN in Pakistan, highlighting disparities in newborn care and the significant influence of modifiable factors including maternal nutrition in determining newborn vulnerability. Such efforts are vital for shaping evidence-based policies for maternal and neonatal health.

## Contributors

ZH conceptualized and secured funding for parent study. ZH, NY, and MK supervised data collection and management. HM,NY, SK,AS, AM, MK were involved in the analysis, interpretation, and writing of the original manuscript. ZH and NY, reviewed and provided scientific revision to the manuscript. All authors agreed prior to submission to take responsibility and be accountable for the contents of the manuscript. All authors read and approved the final manuscript.

## Data sharing statement

The dataset and related materials are available from the corresponding author upon reasonable request.

## Declaration of generative AI and AI-assisted technologies in the writing process

During the preparation of this work the author(s) used ChatGPT 3.5 to improve readability and language of the work. After using this tool/service, the author(s) reviewed and edited the content as needed and take(s) full responsibility for the content of the publication.

## Declaration of interests

The authors declare no competing interests.
