# Supplementary material for: Mapping neonatal vulnerability using the Small Vulnerable Newborn (SVN) framework—secondary analysis of PRISMA Pakistan study
Source: Lancet Reg Health Southeast Asia. 2025 Jan 25;33:100535. doi: 10.1016/j.lansea.2025.100535 (PMC11795797; doi:10.1016/j.lansea.2025.100535)
Supplement: Supplementary Table [file mmc1.docx]

**Table 1: Univariate model for six categories of SVN.**

|  | **Term+SGA**^¥^  **n=461**  **(27·5%)**  Crude RR (95%CI) | **Term+LGA**^¥^  **n=36**  **(2·1%)**  Crude RR  (95%CI) | **PT+AGA**^¥^  **n=210**  **(12·5%)**  Crude RR (95%CI) | **PT+SGA**^¥^  **n=41**  **(2·5%)**  Crude RR (95%CI) | **PT+LGA**^¥^  **n=23**  **(1·4%)**  Crude RR  (95%CI) |
| --- | --- | --- | --- | --- | --- |
| **Age at conception (years)** | 0.98 (0.96-1.00)* | 1.07 (1.02-1.13)* | 1.00 (0.98-1.03) | 0.90 (0.83-0.97)* | 1.03 (0.96-1.10) |
| **No formal**  **education** | 1.01 (0.80-1.27) | 1.32 (0.65-2.66) | 0.99 (0.73-1.34) | 2.04 (0.99-4.21)* | 0.85 (0.37-1.97) |
| **Unemployed** | 0.96 (0.75-1.22) | 0.93 (0.45-1.95) | 0.98 (0.70-1.36) | 1.69 (0.89-3.19)* | 1.01 (0.40-2.55) |
| **Number of previous pregnancies** | 0.85 (0.80-0.90)* | 1.08 (0.95 – 1.24)* | 0.99 (0.93-1.06) | 0.84 (0.72-0.99)* | 0.98 (0.81-1.18) |
| **Bad obstetric history ^^^** | 0.88 (0.68-1.12) | 0.65(0.29-1.44) | 1.07(0.77-1.47) | 0.55(0.25-1.21) | 1.22 (0.51-2.90) |
| **History of tobacco consumption** | 1.32 (1.01-1.71)* | 0.71 (0.29-1.73) | 1.29 (0.91-1.82)* | 1.47 (0.74-2.92) | 0.34 (0.08-1.45)* |
| **Under-nourished**  **(MUAC <23 cm)** | 1.73 (1.35-2.22)* | 0.42 (0.15-1.20)* | 1.16 (0.82-1.64) | 2.89 (1.54-5.45)* | 1.18 (0.46-3.04) |
| **Anemia**  **(Hb <11g/dL)** | 1.00 (0.79-1.28) | 1.18 (0.57-2.42) | 1.21 (0.87-1.69)* | 1.59 (0.77-3.26)* | 0.56 (0.24-1.31)* |
| **HBV/HCV reactivity ^µ^** | 1.01 (0.48-2.12) | 1.20 (0.16-9.23) | 0.42 (0.10-1.77)* | 1.01 (0.13-7.70) | 1.86 (0.24-14.55) |
| **Gestational Hypertension** | 1.17 (0.69-2.02) | 1.38 (0.32-5.96) | 1.42 (0.73-2.78) | 1.85 (0.55-6.28) | 1.07 (0.14-8.12) |
| **Chronic illness ^+^** | 1.18 (0.73-1.91) | 1.66 (0.49-5.61) | 1.11 (0.58-2.12) | 0.46 (0.06-3.39) | 0.83 (0.11-6.29) |

^*^Maternal and delivery characteristics that showed significant associations (p<0.25) at univariate analysis.

^^^ Bad obstetric history: previous miscarriage/stillbirths;

**^+^**Chronic Illness: Hepatitis B Virus; HCV: Hepatitis C Virus; includes known history of diabetes mellitus, chronic hypertension, heart disease, and/or kidney disease.

**^µ^**HBV/HCV: Hepatitis B and C reactivity.
